# Silencing of Glucocerebrosidase Gene in *Drosophila* Enhances the Aggregation of Parkinson's Disease Associated α-Synuclein Mutant A53T and Affects Locomotor Activity

**DOI:** 10.3389/fnins.2018.00081

**Published:** 2018-02-16

**Authors:** Salema B. Abul Khair, Nisha R. Dhanushkodi, Mustafa T. Ardah, Wenfeng Chen, Yufeng Yang, M. Emdadul Haque

**Affiliations:** ^1^Department of Biochemistry, College of Medicine and Health Sciences, United Arab Emirates University, Al Ain, United Arab Emirates; ^2^Institute of Life Sciences, Fuzhou University, Fuzhou, China

**Keywords:** Parkinson's disease, GBA, *Drosophila*, synuclein, neurodegeneration, sleep behavior

## Abstract

**Background:** Mutations in glucocerebrosidase (GBA), a lysosomal enzyme are the most common genetic risk factor for developing Parkinson's disease (PD). We studied how reduced GCase activity affects α-synuclein (α-syn) and its mutants (A30P and A53T) aggregation, neurodegeneration, sleep and locomotor behavior in a fly model of PD.

**Methods:** We developed *drosophila* with GBA gene knockdown (RNAi) (with reduced GCase activity) that simultaneously expresses either wildtype (WT) or mutants such as A30P or A53T α-syn. Western blot and confocal microscopy were performed to study the α-syn aggregation and neurodegeneration in these flies. We also studied the sleep and locomotor activity of those flies using *Drosophila* activity monitor (DAM) system.

**Results:** Western blot analysis showed that GBA RNAi A53T α-syn flies (30 days old) had an increased level of Triton insoluble synuclein (that corresponds to α-syn aggregates) compared to corresponding A53T flies without GBA RNAi (control), while mRNA expression of α-syn remained unchanged. Confocal imaging of whole brain staining of 30 days old *drosophila* showed a statistically significant decrease in neuron numbers in PPL1 cluster in flies expressing α-syn WT, A30P and A53T in the presence GBA RNAi compared to corresponding control. Staining with conformation specific antibody for α-syn aggregates showed an increased number of neurons staining for α-syn aggregates in A53T fly brain with GBA RNAi compared to control A53T flies, thus confirming our protein analysis finding that under decreased GBA enzyme activity, mutant A53T aggregates more than the control A53T without GBA silencing. Sleep analysis revealed decreased total activity in GBA silenced flies expressing mutant A53T compared to both A53T control flies and GBA RNAi flies without synuclein expression.

**Conclusion:** In A53T flies with reduced GCase activity, there is increased α-syn aggregation and dopamine (DA) neuronal loss. This study demonstrates that reduced GCase activity both in the context of heterozygous GBA1 mutation associated with PD and in old age, contribute to increased aggregation of mutant α-syn A53T and exacerbates the phenotype in a fly model of PD.

## Introduction

Parkinson's disease (PD) is a chronic, progressive neurodegenerative disorder typically characterized by the loss of control of movement due to loss of dopaminergic (DA) neurons in the substantia nigra pars-compacta (SNc) (Gibb and Lees, 1991). PD brains shown lesions and intractyoplasmic plaques composed mainly of α-synuclein (α-syn) aggregates. α-syn is a presynaptic protein encoded by the gene SNCA, mutations of which lead to formation of insoluble species of the protein; which in its native state is monomeric and soluble. α-Syn is also reported to exist as helically folded tetramer that resist aggregation (Bartels et al., 2011). PD correlates with the formation of insoluble fibrillar aggregates in the central nervous system that contain α-syn (Braak and Braak, 2000). The misfolding and aggregation of α-syn can be aggravated by point mutations in the SNCA gene. A53T and A30P mutations (mutations that cause autosomal dominant form of PD; Polymeropoulos et al., 1997; Krüger et al., 1998) alter the fibrillation characteristics of α-syn. These mutations including others associated with familial PD (A30P, A53T, and E46K) have an increased aggregation propensity *in vitro*, similar to *in vivo* aggregation seen in fibrillar Lewy bodies (Conway et al., 2000a,b; Greenbaum et al., 2005).

GBA encodes for β-glucocerebrosidase (GCase) enzyme; a lysosomal enzyme that hydrolyzes the glycolipid glucosylceramide (GlcCer). GBA mutations cause mild to severe forms of Gaucher's disease (GD), wherein due to low enzymatic activity, there is a buildup of the substrate of GBA enzyme, GlcCer in the organs. The association between GBA mutations and PD was initially discovered clinically in patients with PD, although noted rarely in patients with GD, showed up more frequently in their relatives who were heterozygous for the mutation (Goker-Alpan et al., 2004). Subsequent larger studies confirmed patients with PD and associated Lewy body disorders had an increased frequency of having GBA1 mutations (Sidransky, 2005). The frequency of GBA1 mutation in PD patients is estimated to be between 5 and 10%, and the penetrance and lifetime risk of developing PD for these mutation carriers is estimated to be 20% at the age of 70 years (Sidransky and Hart, 2012; Schapira, 2015). This association can be due to mutations in GBA that promotes α-syn aggregation or substrate accumulation due to loss-of-function of enzyme activity, which affects α-syn processing and clearance. GBA loss of function might thus cause substrate accumulation and can affect the autophagy-lysosome pathway (ALP) (Sidransky and Lopez, 2012). GBA mutation is known to cause a positive feedback loop causing α-syn accumulation that further prevents trafficking of GBA to lysosomes (Mazzulli et al., 2011). Inhibition of GBA enzyme in mice and in *in vitro* model shows an increased accumulation of α-syn and the lipids glucocerebroside and glucosylsphingosine due to impaired lysosomal function (Mazzulli et al., 2011). Studies addressing the pathological mechanisms underlying GBA associated Parkinsonism will help in designing treatment strategies for Parkinsonism in patients with GBA1 mutation. Current therapies like enzyme replacement and substrate reduction therapy with the iminosugar miglustat has little or no effect on the progression of Parkinsonism in patients with GBA1 mutation (Kraoua et al., 2011). With the advent of glucocerebrosidase for gene therapy and molecular chaperones (that increase GCase activity) with beneficial effects on induced pluripotent stem cells (iPSc) derived dopaminergic neurons, the molecular mechanisms involved in the protective role of GCase in α-syn toxicity still remains intriguing (Rocha et al., 2015; Aflaki et al., 2016).

Decreased GCase activity has been reported in patients with or without GBA (Murphy and Halliday, 2014; Murphy et al., 2014; Aflaki et al., 2016) as there is an age-dependent reduction in GCase activity that may lower the threshold to develop PD in persons even in the absence of any genetic mutation in GBA gene. Several *in vitro* and *in vivo* experimental evidences suggest a correlation between this decreased activity and accumulation of α-syn (Cullen et al., 2011; Mazzulli et al., 2011; Sardi et al., 2011; Gegg et al., 2012). Reduced GCase activity is seen in brain regions with α-syn accumulation, and, reduced leukocyte GCase activity is linked to increased plasma levels of α-syn oligomers (Gegg et al., 2012; Nuzhnyi et al., 2015; Barkhuizen et al., 2016). Simultaneous mutations in both GBA and SNCA gene has been shown to aggravate the PD phenotype in a transgenic mice that harbors the GBA mutation (p.L444P) that leads to 40% reduction in GCases enzyme activity when co-expressed with the SNCA A53T mutation. It is also showed exacerbated motor and gastrointestinal symptoms in comparison to mice expressing only the A53T mutation (Fishbein et al., 2014). It is shown that deletion of dGBA1b with reduced GCase activity has an increased aggregation and neurodegeneration *per se*, and α-syn expression did not enhance the phenotype (Davis et al., 2016). While few studies in *drosophila* examined the effect of reduced GCase activity on WT α-syn alone (Suzuki et al., 2015), we were interested to explore the effect of GBA silencing on the metabolism and toxicity of wildtype (WT), A30P and A53T mutants of SNCA in *drosophila*. This was achieved by silencing dGBA using RNAi construct, while simultaneously expressing α-syn (WT/A30P/A53T) with GMR-Gal4 or TH-Gal4 drivers. The effect of GBA silencing on WT and mutant α-syn metabolism and toxicity was studied by observing α-syn aggregation and neurodegeneration (by means of western blot and confocal microscopy), and behavioral consequences like fly sleep and locomotor activity (using *drosophila* activity monitor and negative geotaxis assay). Earlier study in *drosophila*, although shown an increased α-syn mutant A53T aggregation during GBA1b silencing, however, did not report on enhancement of phenotypes like shortened life-span, climbing defects, sensitivity of mechanical or heat stress of GBA1b mutants (Davis et al., 2016). Our current study demonstrates that reduction of GCase activity comparable to heterozygous GBA1 mutation associated with PD contributes to increased aggregation of mutant α-syn A53T, causes enhanced DA neuronal loss and exacerbates the motor as well as non-motor phenotypes in a fly model of PD.

## Materials and methods

### Fly stock and maintenance

The following fly stocks used in the experiment were obtained from Bloomington *Drosophila* Stock Center: UAS-h[WT]αSyn (8146), UAS-h[A30P]αSyn (8147), UAS-h[A53T]αSyn (8148), GMR-Gal4 (8121), Th-Gal4 (8848) and Cyo;TM2/TM6b (3604). The following transgenic flies for UAS-GBA-RNAi were obtained from Vienna *Drosophila* Resource Center (VDRC): UAS-dGBA1b (*drosophila* homolog of GBA1) -two clones 21337 and 101212, both targeting dGBA (CG31414). For targeted tissue specific transgene expression, we used the UAS/Gal4 system (Brand and Perrimon, 1993). GMR-Gal4 or TH-Gal4 driver lines (Friggi-Grelin et al., 2003) were crossed with UAS-transgene flies, to downregulate dGBA (using RNAi) and/or to overexpress WT α-syn/A30P/A53T mutants. Flies were routinely kept at 25°C (unless specified) on cornmeal yeast agar medium using a 12 h light and 12 h dark (LD) cycle. Male flies were used for all experiments except for RT-PCR and GCase assay.

### GCase activity assay

Fly heads with silenced dGBA under control of GMR-Gal4 or control fly were homogenized in 50 mM phosphate buffer, pH 7.0 with protease phosphatase inhibitor and centrifuged at 15,000 rpm, for 20 min at 4°C. Samples supernatant were then collected in fresh tubes and BCA assay was performed for protein quantification. On a 96 well plate, 20 μl of distilled water was loaded to each well. Then 200 μl of distilled water was added to one of these wells and to the other well 200 μl of calibrator from the β-glucosidase assay kit (Abnova, Taiwan) was added following the protocol provided in the kit. Twenty microliter of sample containing equal amount of protein was loaded into other wells. To the sample wells 200 μl working reagent was added, plate was tapped briefly to mix. An initial reading was taken at 405 nm (*t* = 0) and after 20 min of incubation, final reading (*t* = 20) was taken using a microplate reader (Victor X3 2030, Perkin Elmer). Results were calculated as mentioned in the protocol provided by the manufacturer and finally expressed as percentage of control (GMR/GMR). Results are presented as Mean ± S.E.M of three independent experiments.

### RT-PCR

Total RNA was isolated from female fly heads at 0 and 30 days using TRIzol (Life Technologies), followed by cDNA synthesis using the GoSCRIPT reverses transcription system (Promega). PCR was performed using the following primers specific for α-syn, dGBA and RP49 in Veriti 96 well Thermal cycler (Applied Biosystems):

α-syn forward: 5′- TGT AGG CTC CAA AAC CAA GG-3′
α-syn reverse: 5′- TGT CAG GAT CCA CAG GCA TA-3′
dGBA forward: 5′- GGT GAT TAG CTC CAG CAA GGA-3′
dGBA reverse: 5′- CAG CGC CCA TGA TTT TCA GAG-3′
RP49 forward: 5′- CCG CTT CAA GGG ACA GTA TC-3′
RP49 reverse: 5′-TCT CCT TGC TTC TTG GAG GA-3′

For densitometric analysis of RT-PCR for dGBA, values were normalized with RP49. For statistical analysis and comparison of GBA mRNA levels between α-syn species and its corresponding dG2/dG2 α-syn were carried out considering α-syn as 100% to derive the value of its corresponding dG2/dG2 α-syn. Additionally, GBA mRNA in GMR/+ is taken as 100% to calculate the value of dG2/dG2;GMR/+.

### Climbing assay

Startle induced climbing assay was performed according to a previously described protocol (Feany and Bender, 2000). Ten male flies were placed in graduated empty vials and allowed to recover for 10 min. Fly vials were then firmly tapped three times, number of flies that crossed vertical movement of 6 cm in 10 s calculated. This was repeated for five trials at 20 s intervals and the climbing index represented as percentage of flies (the number of flies crossed divided by total number of flies multiplied by 100). The mean index of five trials were calculated and presented as Mean± S.E.M of three independent experiments. Decline in climbing activity with age was calculated for old flies (20 and 30 day) in each genotype by normalizing with the climbing index of 0 day young flies in the corresponding genotype. For example, decline in climbing Index at 20 day = climbing Index at 20 day/climbing index at 0 day X 100.

### Immunoblotting

For whole head lysates, male fly heads were homogenized in (5 μl per head) tissue lysis buffer (Sigma Aldrich) containing protease/phosphatase inhibitor and EDTA 1mM. Samples were then centrifuged at 6,000 rpm for 20 min at 4°C and supernatant were collected in fresh tubes. Samples were then mixed with 5X reducing sample loading dye and boiled for 5 min at 95°C, cooled down, briefly spun and then separated on SDS-PAGE gels (12%). For isolation of triton-soluble and insoluble fractions, we followed the protocol as described earlier (Davis et al., 2016). Fly heads were homogenized (5 μl per head) in triton lysis buffer (50 mM Tris-HCl, pH 7.4, 1% Triton X-100, 150 mM NaCl, and 1 mM EDTA containing protease phosphatase inhibitor, centrifuged at 14,000 rpm for 20 min at 4°C, and the supernatants were collected as the triton-soluble fractions. The pellets were suspended in 1X laemmli buffer (2% SDS, 2% 2-Mercaptoethanol, 10% glycerol, 0.002% bromophenol blue, 100 mM Tris-Hcl pH6.8), heated for 10 min and then the supernatant collected as triton-insoluble fraction. BCA assay was carried out to determine the protein content in triton-soluble fraction before adding equal volume of 2X laemmli buffer. The extracts containing equal amount of proteins were electrophoresed in 4–12% gradient acrylamide gels (Bio-Rad) and the proteins were transferred to PVDF membrane at 90 V for 90 min. The membranes were boiled in PBS for 5 min, followed by blocking in 5% non-fat milk in PBS-Tween 20 (0.05% Tween-20) and probed overnight at 4°C with 1:3000 dilution of mouse monoclonal anti-α-syn (Syn-1) (BD Biosciences). The membranes were washed several times with PBS-T and incubated with 1:80,000 dilution of HRP-conjugated goat anti-mouse antibody for 1 hr at room temperature. After three washes in PBS-T for 30 min, protein bands were then visualized using Super Signal West Femto Chemiluminescent Substrate Kit (Pierce, Rockford, USA). Band intensities were quantified using Image J (NIH) software and values are represented as percentages. Results are presented as Mean± S.E.M of three independent experiments. For densitometric analysis of α-syn blots, values were normalized to corresponding β-actin. The ratio of triton Insoluble /Triton-soluble was done by normalizing densitometric values of Triton-Insoluble α-syn to corresponding triton-soluble α-syn (Triton-Insoluble/Triton-Soluble X 100). For statistical analysis and comparison between α-syn and corresponding dG2/dG2 α-syn noramalization was carried out considering α-syn as 100% to derive the value of its corresponding dG2/dG2 α-syn.

### Sleep and locomotor activity assays

Flies were maintained in a LD cycle (12h: 12h) cycle at 25°C with equal population densities. Locomotor activity and sleep behavior were recorded from single males using the *Drosophila* Activity Monitoring (DAM) system (Trikinetics, Waltham, USA) as described (Joiner et al., 2006; Pitman et al., 2006). The DAM monitor contains 32 channels, each connected to a small glass tube containing each fly, in which the movement of individual flies can be monitored, as they break the infrared beam that bisects the tube. Movements were recorded in 1 min bin size using DAM 308 software. Sleep in *drosophila* is defined as a bout of 5 or more minutes of inactivity (Shaw et al., 2000). The average length of a sleep bout was calculated as the total amount of sleep (min) divided by the number of sleep bouts for the day and night. The activity index refers the activity when flies were awake (number of recorded movement divided by the total time (min). Single males were individually recorded (16 flies per genotype) using the DAM system and three independent experiments were performed for each genotype. With data extracted using DAM filescan110, sleep behavior analysis was done using Microsoft Excel.

### Immunohistochemistry and confocal microscopy

Brains of different groups from 30-day old flies were dissected in cold PBS, and fixed in 4% paraformaldehyde in PBS for 1 hr. Samples were washed and permeabilized in 0.3% Triton X-100 in PBS (Wash buffer) overnight. Fixed brains were stained with mouse anti-Tyrosine Hydroxylase (TH) antibody (Mouse monoclonal, Immunostar)/ anti-α-syn antibody (Syn1- Rabbit polyclonal, BD biosciences)/anti- α-syn filament antibody (Rabbit polyclonal, Abcam) at 1:200 dilution for 48 hr. After incubation, brains were washed thrice, and incubated with corresponding secondary antibodies overnight (anti-mouse Alexa 488/anti-rabbit Alexa 594) at 1:1,000 dilution. After thorough washing, stained brains were mounted using Fluoromount mounting medium (Sigma) and images were acquired in confocal microscope (Nikon EZC1). DA neuron clusters were analyzed as previously described (Whitworth et al., 2005). Optical sections of brains were acquired at.40-μm intervals using a 40X objective for whole-brain imaging. Confocal stacks were merged into a single plane by using Nikon EZC1 software. The number of TH-positive neurons within the PPL1 dopaminergic neuron cluster was counted by visual inspection of individual confocal Z-series images. An average of eight brains was analyzed in both the hemisphere for each genotype and the result expressed as Mean ± S.E.M.

## Results

### Targeted silencing of *Drosophila GBA* gene using RNAi results in reduced mRNA of GBA1 & GCase activity which causes eye defects

**Figure 1:**
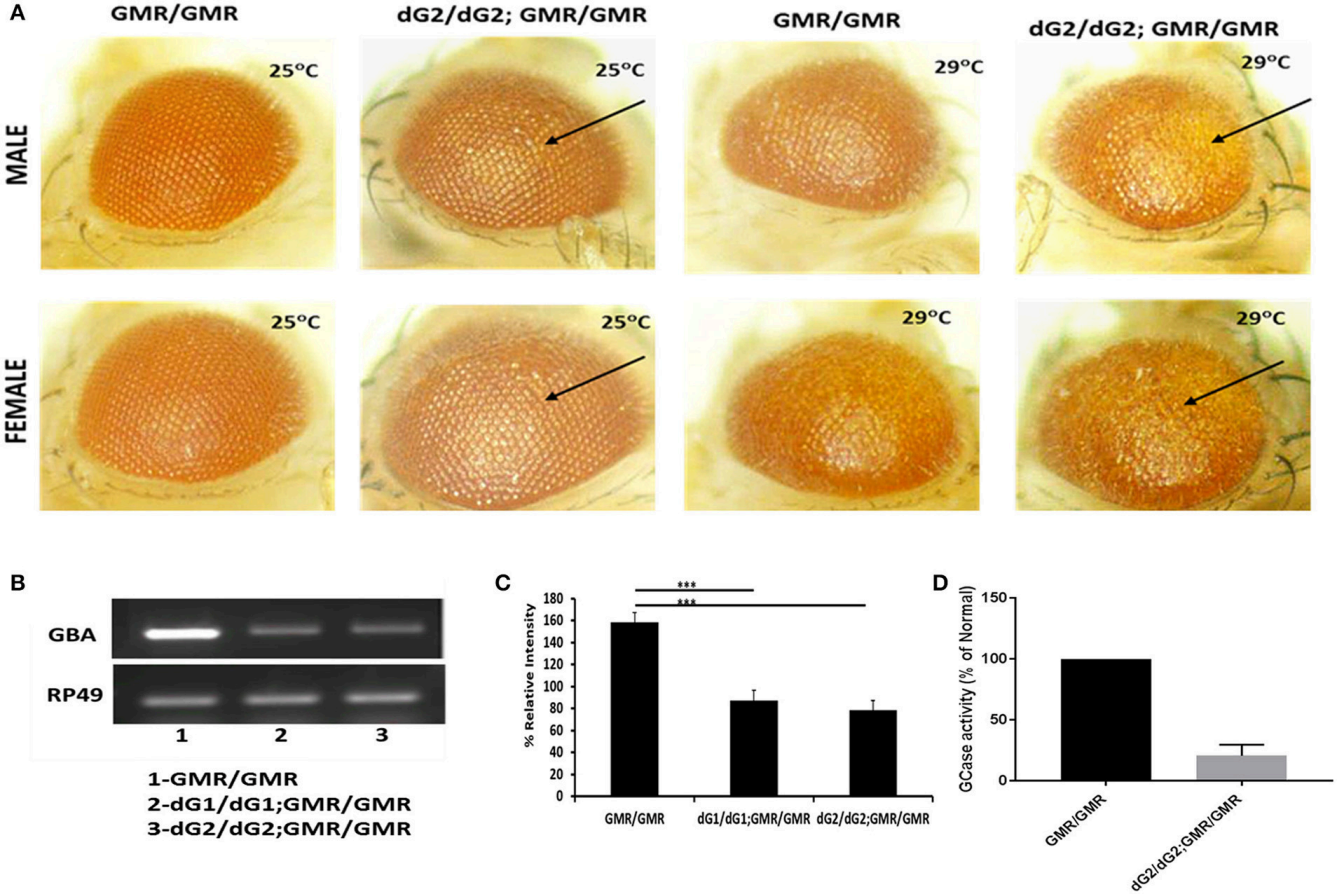
Targeted silencing of *drosophila* GBA gene using RNAi (dG2/dG2). **(A)** Eye expression shows phenotypic effect in male and female flies maintained at 25° and 29°C carrying two copies of dG2 (GBA RNAi) with GMR-Gal4 driver compared to two copies of GMR-Gal4 alone. **(B)** RT-PCR results showing that there is a significant downregulation of GBA in fly lysates of dG1/dG1 and dG2/dG2 (two different clones targeting CG13414, fly homolog of GBA1) under control of GMR-Gal4 driver compared to corresponding GMR-Gal4 control and the corresponding graph shown in **(C)**. On doing other preliminary screenings, we decided to continue this study with dG2/dG2. **(D)** GCase enzyme activity assay was performed to confirm silencing of GBA enzyme in flies with two copies of dG2 in the presence of two copies of GMR-Gal4 driver. The enzyme activity is expressed as percentage of control. Graph represents mean ± S. E. M for 3 independent experiments. Differences in means compared by Students *t*-test ^***^ represents *p* < 0.0001.

Two different clones of transgenic GBA RNAi fly lines (named as dG1/dG1 and dG2/dG2) with inverted repeat RNA targeting the *drosophila* homolog of the GBA1 gene, namely, CG31414 (dGBA1b) that has 31% homology to human GBA1 gene were used under the control of UAS-Gal4 system. DGBA1a is shown to express in midgut whereas dGBA1b is expressed throughout body especially in the larval and adult brain. Several studies have shown a successful downregulation of GCase activity by targeting CG31414 (Suzuki et al., 2015; Davis et al., 2016). Down-regulation of GBA1b in *drosophila* eye shows phenotypic effect in flies with two copies of dGBA1 RNAi (dG2/dG2) under control two copies of GMR-Gal4 driver when compared to corresponding GMR-Gal4 alone (Figure 1A). To further confirm that the eye defect observed in those flies is due to GBA silencing, we performed RT-PCR and GCase assay. RT-PCR results showed a significant downregulation of GBA1 in fly eye samples of dG1/dG1 and dG2/dG2 (both targeting CG31414) in the presence of GMR-Gal4 compared to corresponding GMR-Gal4 control (Figures 1B,C). Although our eye screening and mRNA knockdown revealed no significant variation between dG1/dG1 and dG2/dG2, we decided to continue the current study with dG2/dG2 *drosophila* lines. We further performed GCase enzyme activity assay to confirm silencing of GBA1 enzyme in flies with two copies of dG2/dG2 in the presence of two copies of GMR-Gal4 driver (Figure 1D). Thus we obtained flies with ~70% reduction in GCase activity in the heads. Since our experimental flies carry only single copy of GMR-Gal4 driver, we anticipate ~50% reduction of GCase activity in these flies and thus they represent as model for heterozygous GBA1 mutations associated with PD. To study the effect of GBA silencing on neurotoxicity of α-syn and its mutants, we used UAS-Gal4 bipartite system to create transgenic flies with single copy of tissue specific Gal4 driver bearing two copies of GBA RNAi (dG2/dG2) simultaneously in the presence or absence of α-syn wild-type (WT) and mutants (A30P/A53T). We did not observe eye phenotypic defects in these flies expressing single copy of GMR-Gal4 driver with/without dG2/dG2 in the presence of α-syn (Figure S1).

### Silencing of GBA1 does not affect α-syn mRNA transcription and protein synthesis

**Figure 2:**
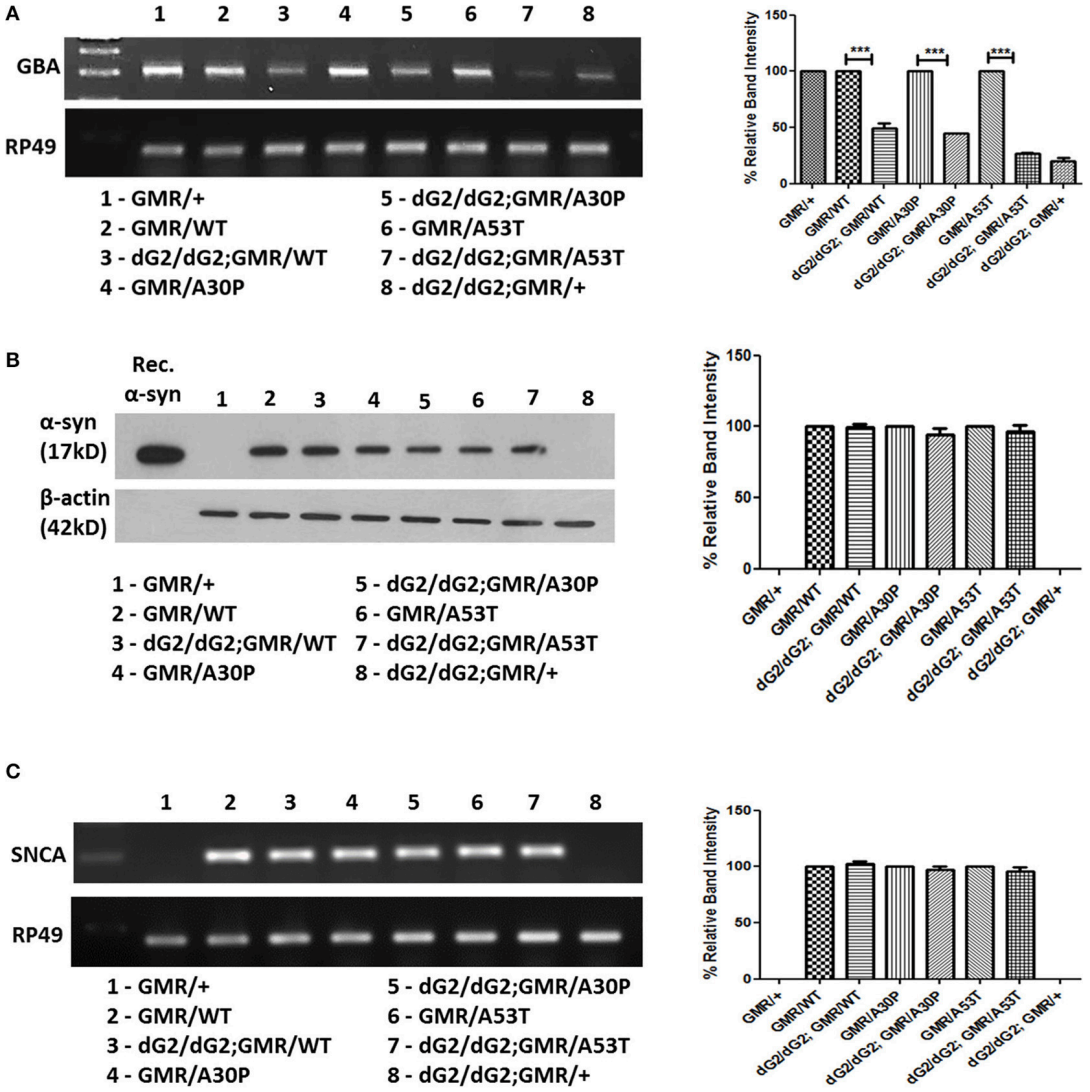
Absence of transcriptional changes in α-syn expression in dG2/dG2 RNAi flies. **(A)** RT-PCR confirms knockdown of GBA1 mRNA in flies with dG2/dG2 (in presence/absence of α-syn-WT, A30P, A53T) with single copy of GMR-Gal4 driver compared to respective control flies. This confirms significant transcriptional downregulation of GBA even in the absence of eye phenotype (in flies with single copy of GMR). Corresponding densitometric analysis of RT-PCR was done by normalizing controls to 100% and experimental flies (dG2/dG2 with/without α-syn) are compared against their respective controls. **(B)** The effect of GBA silencing on α-syn protein expression, immunoblotting was done by whole tissue lysis extraction method. Further densitometric analysis revealed no change in α-syn protein level in flies with dG2/dG2 expressing α-syn (WT, A30P, and A53T) under GMR-Gal4 drive compared to control flies expressing α-syn (WT, A30P, and A53T) alone. Corresponding densitometric graph where controls (GMR with/without α-syn) normalized to 100% is shown. **(C)** RT-PCR results show no changes in the transcriptional level of α-syn during GBA silencing. In densitometric analysis of α-syn RT-PCR, controls (GMR with/without α-syn) are normalized to 100% to compare experimental flies (dG2/dG2 with/without α-syn) against their respective controls. Graph represents mean ± S.E.M. for 3 independent experiments. Differences in means were compared by one-way ANOVA followed by the Newman-Keuls Multiple Comparison *post hoc* test. ^***^ represents *p* < 0.0001.

In spite of absence of marked eye phenotype in flies expressing single copy of GMR-Gal4 driver with dG2/dG2, there was a marked down-regulation of GBA1 mRNA level as confirmed by RT-PCR (Figure 2A). Immunoblot assay using whole tissue lysis extraction method and further densitometric analysis, we observed no change in α-syn protein level in flies with dG2/dG2 expressing α-syn (WT, A30P, A53T) under control of GMR-Gal4 driver compared to control flies expressing only α-syn (WT, A30P, A53T) at 10 days (Figure 2B). To further know whether knockdown of GBA1 affects α-syn transcription, corresponding fly samples were used to examine the effect of GBA1 silencing on the α-syn mRNA level. Densitometric analysis of RT-PCR in which controls (GMR with/without α-syn) are normalized to 100% and experimental flies (dG2/dG2 with/without α-syn) were compared against their respective controls is shown in Figure 2C. As expected, there was no transcriptional change in α-syn expression when GBA1 is silenced.

### Increased **α**-syn aggregation in triton X-insoluble fraction in dG2/dG2 RNAi flies expressing A53T mutant synuclein

**Figure 3:**
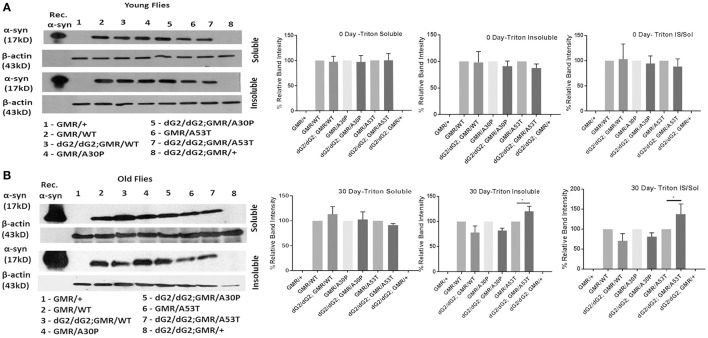
Triton-soluble and triton-insoluble α-syn level in dG2/dG2 RNAi flies expressing WT, A30P, and A53T synuclein. Western blot of triton-soluble and insoluble fraction of 0 day **(A)** and 30 day **(B)** old fly head lysates probed for α-syn showed increase in triton-insoluble α-syn levels in dG2/dG2 A53Tmutant flies compared to corresponding A53T control flies at 30days. In the corresponding densitometric analysis, controls (GMR with/without α-syn) are normalized to 100% and experimental flies (dG2/dG2 with/without α-syn) are compared against their respective controls. Triton-Insoluble α-syn was also normalized to corresponding triton-soluble α-syn. Graph represents mean ± S.E.M. for 3 independent experiments. Differences in means were compared by one-way ANOVA followed by the Newman-Keuls Multiple Comparison *post hoc* test. ^*^ represents *p* < 0.05.

Understanding the α-syn aggregation propensity of WT and mutant α-syn protein under GBA1 silencing will be useful in determining the role of GBA in the toxicity of α-syn in these flies. To clarify further and to find out the fate of α-syn protein in flies when GBA is silenced, we have processed the samples into triton-soluble and insoluble fractions. It is noteworthy that Triton- insoluble fraction is considered to contain aggregated form of α-syn protein. In western blot of triton-soluble and insoluble fraction of 0 day (Figure 3A) and 30 day (Figure 3B) old fly head lysates probed for α-syn, controls (GMR flies with α-syn and without dG2/dG2) were normalized to 100% and experimental flies (GMR flies with α-syn and dG2/dG2) were compared against their respective controls. At 0 days, there was little or no change in soluble α-syn in WT, A30P and A53T dG2/dG2 flies and an insignificant decrease in triton-insoluble α-syn was seen in A30P dG2/dG2 and A53T dG2/dG2 flies compared with respective controls. There was a slight decrease in triton-insoluble α-syn in WT α-syn flies at 30 days (not statistically significant) when GBA1 is silenced. In A30P α-syn expressing 30 day old flies with GBA1 silencing, there was not a significant change in α-syn level. Interestingly, there was a decrease in triton-soluble and a significant increase in triton-insoluble α-syn level in dG2/dG2 A53T mutant flies compared to corresponding A53T control flies at 30 days. We observed a clear trend of increased aggregation in A53T mutant flies with GBA silencing compared to its control A53T flies and this trend is not observed in WT and A30P flies.

### Increased neurodegeneration in dG2/dG2 flies expressing WT, A30P, and A53T synuclein

**Figure 4:**
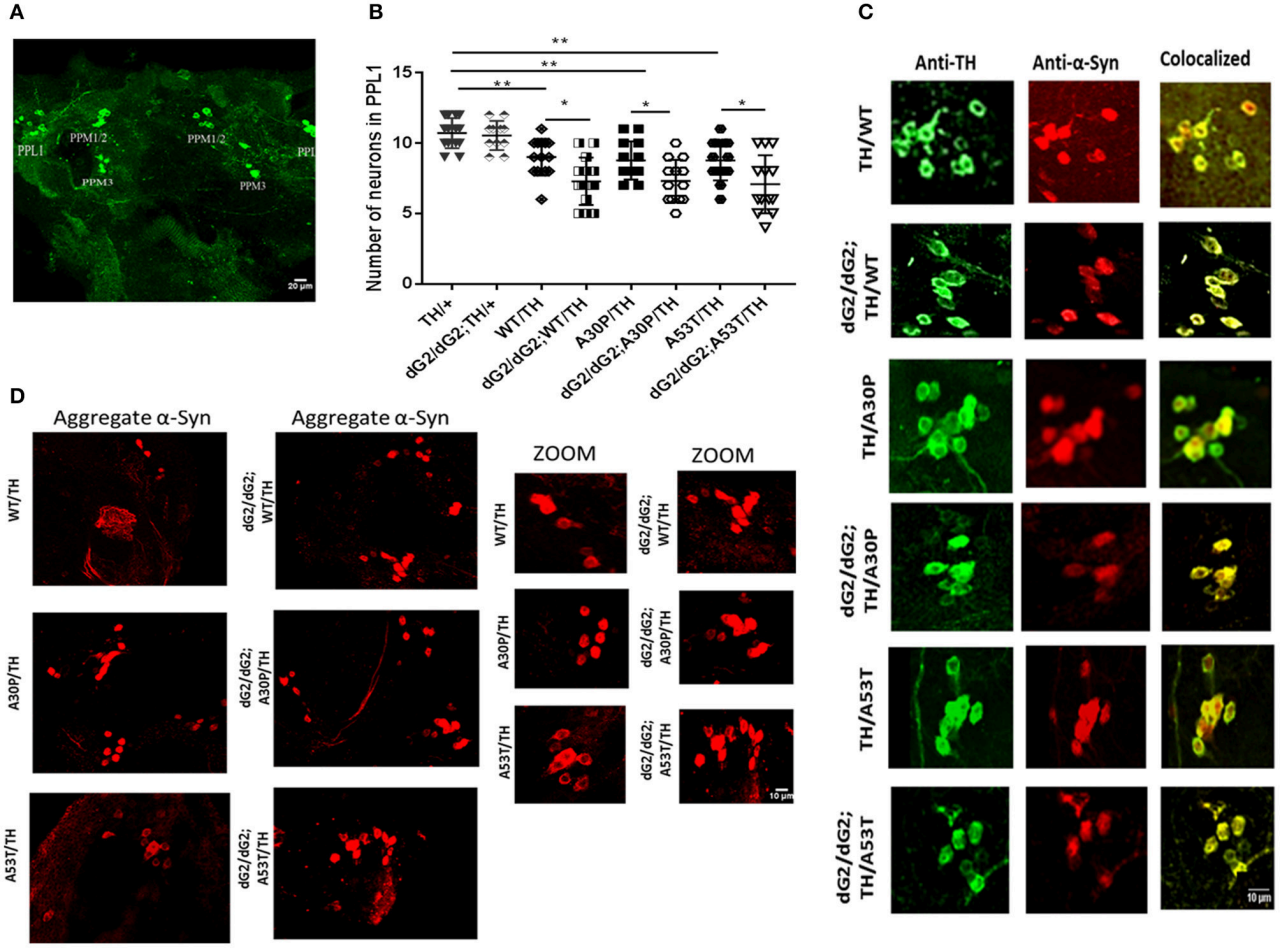
Increased neurodegeneration in dG2/dG2 flies expressing WT, A30P, and A53T synuclein. **(A)** Representative image of a projected Z-series of 30 day-old control fly brain with TH-Gal4 driver stained with anti-Tyrosine Hydroxylase (TH) to identify dopaminergic (DA) neurons. DA neurons within each cluster are indicated by labels. **(B)** Relative number of DA neurons within the PPL1 cluster of 30 day-old dG2/dG2 RNAi flies under control of TH-Gal4 driver expressing WT, A30P, A53T synuclein (*n* = 16), compared to age-matched corresponding control flies without dG2/dG2 RNAi (*n* = 16). There was also significant decrease in number of DA neurons in flies expressing α-syn (WT/A30P/A53T) compared to control TH/+ flies. Differences in means were compared by one-way ANOVA followed by the Newman-Keuls Multiple Comparison *post hoc* test. ^**^ represents *p* < 0.001, ^*^p < 0.05. **(C)** Representative image of projected Z-series of PPL1 cluster in 30 day-old fly brain with under control of TH-Gal4 driver co-stained with anti-TH (green) and anti-syn antibody (red) confirms expression of α-syn in DA neurons in different genotypes. **(D)** Representative immunofluorescent staining of 30 day-old fly brain expressing WT, A30P, and A53T synuclein (TH-Gal4 UAS transgenes) with/without GBA silencing stained with conformational antibody (anti-α-synuclein filament antibody) for α-syn aggregates.

Next we performed immunohistochemical analysis to examine whether silencing of GBA exacerbate the synuclein toxicity in our flies. We stained TH neuron clusters in GBA1 silenced fly brains under control of TH-Gal4 driver to understand the role of GBA1 on neurodegeneration. GBA1 silencing has been shown to increase neurodegeneration in the presence of α-syn expression (Suzuki et al., 2015; Davis et al., 2016) and we further would like to confirm this finding in our flies. Figure 4A shows a representative image of a projected Z-series of 30 day-old fly brain under control of TH-Gal4 driver (with/without dG2/dG2 RNAi) stained with anti-Tyrosine Hydroxylase (TH) to label dopaminergic (DA) neurons. We chose to count TH neuron number in PPL1 (posterior protocerebral lateral 1) cluster as previous reports have shown a decreased neuron number in these clusters (Davis et al., 2016). Relative number of DA neurons within the PPL1 cluster of 30 day-old dG2/dG2 RNAi flies with TH-Gal4 UAS transgenes expressing WT, A30P, A53T synuclein (*n* = 16), compared to age-matched corresponding control flies without dG2/dG2 RNAi (*n* = 16) is shown in Figure 4B. There was also significant decrease in number of DA neurons in flies expressing α-syn (WT/A30P/A53T) compared to control TH/+ flies without α-syn expression. Similar to previous reports our results show a profound decrease in neuron numbers in PPL1 cluster in GBA RNAi flies compared to respective controls (Fishbein et al., 2014; Davis et al., 2016). To confirm expression of α-syn in TH neurons in our experimental flies under TH-Gal4 driver, we co-stained for TH neurons and α-syn. Figure 4C shows representative image of projected Z-series of PPL1 cluster in 30 day-old fly brain with TH-Gal4 UAS transgenes co-stained with anti-TH (green) and anti-syn antibody (red) confirms expression of α-syn in DA neurons in different genotypes. Using a conformational antibody for α-syn, that stains only α-syn aggregates, we wanted to confirm our western blot finding that there was an increase in A53T aggregation under GBA silencing. Representative immunofluorescent staining of 30 day-old fly brain expressing WT, A30P, and A53T synuclein (under control of TH-Gal4 driver) with/without GBA silencing and stained with conformational antibody for α-syn aggregates is shown in Figure 4D. We found a gross increase in number of neurons staining for α-syn aggregates in GBA silenced fly brains expressing A53T compared to A53T fly brains without GBA silencing.

### Negative geotaxis assay in dG2/dG2 flies expressing A53T α-synuclein mutant

**Figure 5:**
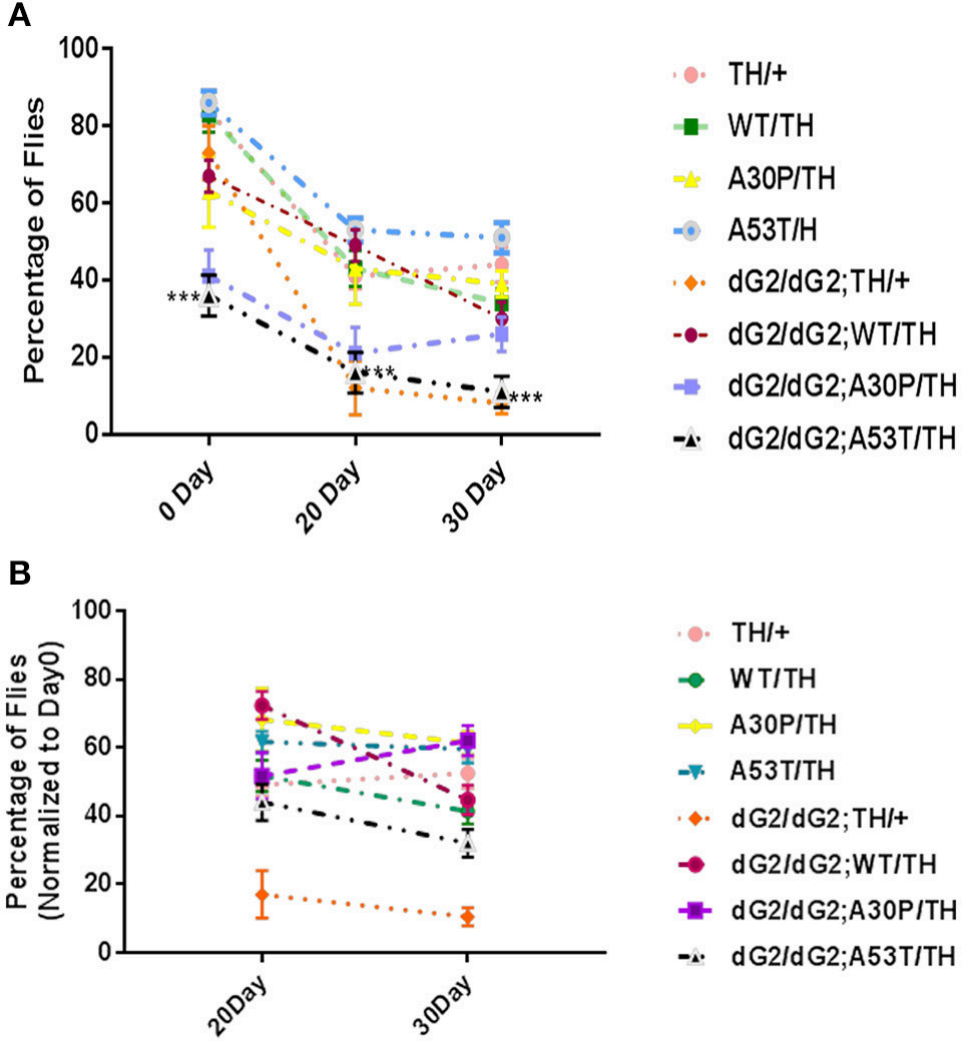
Decreased activity of dG2/dG2 flies in negative geotaxis assay. **(A)** Negative geotaxis assay revealed a decreased movement in dG2/dG2; A53/TH compared to A53T/TH and dG2/dG2 control flies at Day0. **(B)** Climbing Index of old flies (20 and 30 day) normalized to 0 day young flies for each genotype showed a significant decline in activity with age in case of control dG2/dG2 flies. The dG2/dG2 A53T flies did not show prominent decline compared to dG2/dG2 control because the movement is severely compromised in these flies even at day 0. Data represents Mean + S. E. M of 3 independent experiments. Multiple comparisons between means were done by one-way ANOVA followed by Bonferroni correction. Comparison was done between TH/+ vs. dG2/dG2;TH/+, dG2/dG2 vs. dG2/dG2;WT/TH, dG2/dG2; A30P/TH and dG2/dG2;A53T and WT/TH vs. dG2/dG2;WT/TH and A30P vs. dG2/dG2;A30P/TH and A53T vs. dG2/dG2;A53T/TH). ^***^ represents *p* < 0.0001.

Climbing assay (negative geotaxis) is widely used to assess locomotor behavior in flies. Climbing assay was conducted on GBA RNAi flies to assess the effect of reduced GCase activity on locomotor response when startled. At 0 Day dG2/dG2 a53T flies show significant decrease in climbing activity compared to both A53T/TH/ and dG2/dG2;TH/+ control flies (Figure 5A). 30-day old flies revealed a decreased movement when GBA is silenced with dG2/dG2 compared to the corresponding flies expressing A53T synuclein without GBA silencing in the presence of TH-Gal4 driver. However, we noticed that there was also a decrease in locomotor activity in GBA silenced control flies not expressing synuclein. The decline in climbing index with age was most pronounce in dG2/dG2 control flies (Figure 5B). Compared to dG2/dG2 control flies, the decline in climbing activity with age was less pronounced in dG2/dG2 A53T flies since even young dG2/dG2 A53T flies show decreased climbing ability. It is not clear at this stage the reason for the decreased locomotion showed by GBA RNAi fly. Recent report also found abnormal climbing ability and sleep with mutant *drosophila* GBA gene supporting our result (Whitworth et al., 2005). Additionally, our results also corroborate previously reported study in flies where GBA1b deletion resulted in decreased locomotor activity due to reduced GCase activity *per se* and α-syn expression did not aggravate this phenotype (Davis et al., 2016).

### Sleep behavior in dG2/dG2 RNAi flies expressing wildtype and mutant α-synuclein

**Figure 6:**
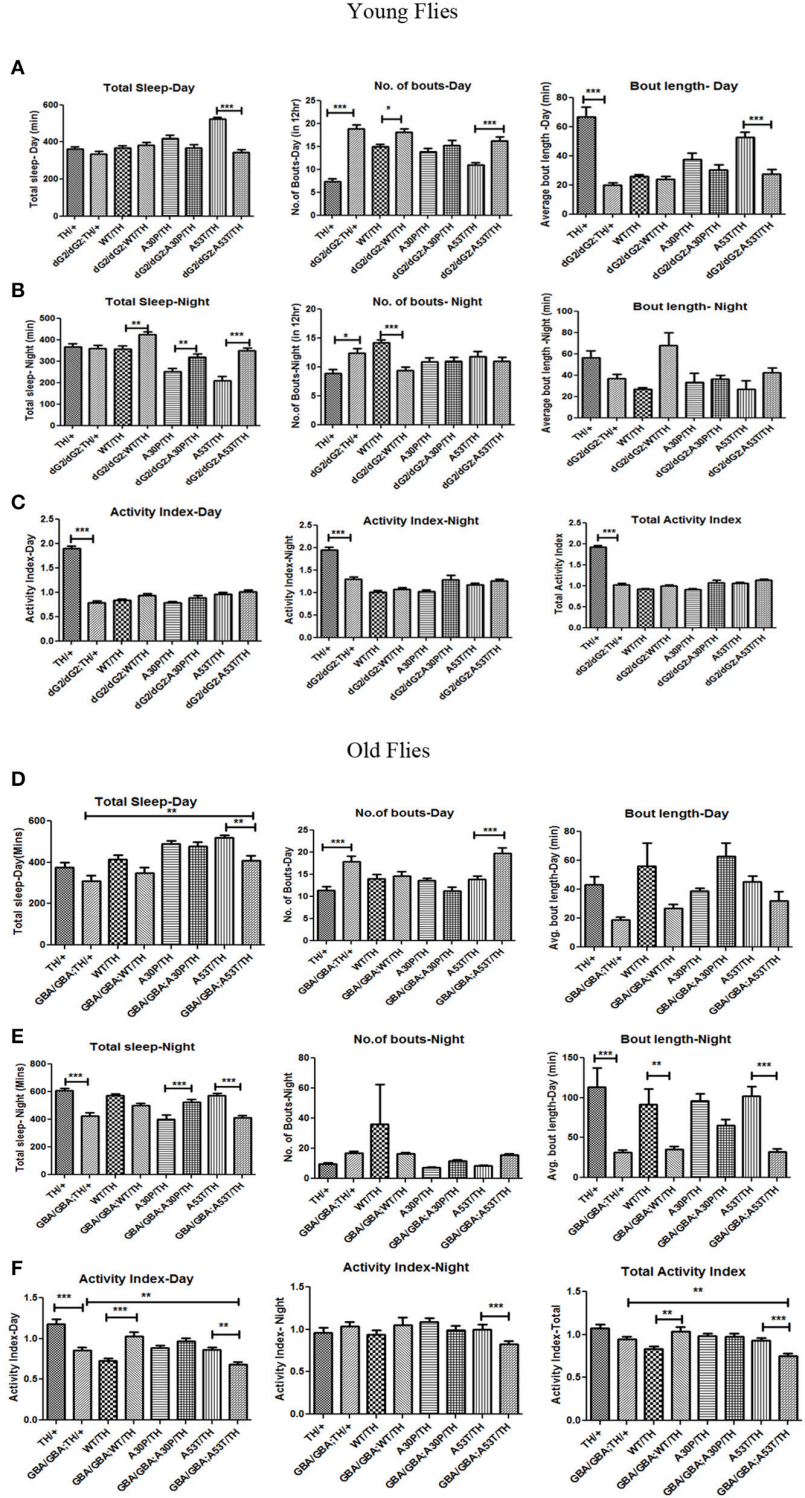
Sleep Behavior in dG2/dG2 GBA RNAi flies expressing wildtype and mutant α-synuclein. Sleep behavior in young flies (0–3days) with and without dG2/dG2 silencing, expressing wildtype (WT), A30P and A53T mutant synuclein using TH-Gal4 is represented as total length of sleep, fragmentation in sleep indicated by sleep bout number, and the average length of sleep in 12 h light (Day) and 12 h dark (Night) cycles **(A,B)**. Activity index represents fly activity level during the wake periods in day, night and in total **(C)**. GBA silenced A53T old flies showed pathogenic symptoms such as increased daytime sleep, with an increased number of bouts **(D)** and a decreased night sleep with decreased night bout length **(E)**. These flies also show marked reduction in wake activity during day, night and in total **(F)**. Bars represent mean values of at least three independent experiments ± the standard error of the mean from 16 flies that were individually recorded in each experiment conducted using *drosophila* activity monitor. Differences in means were compared by one-way ANOVA followed by the Newman-Keuls Multiple Comparison *post hoc* test (between TH/+ vs. dG2/dG2;TH/+, dG2/dG2 vs. dG2/dG2;WT/TH, dG2/dG2; A30P/TH and dG2/dG2;A53T and WT/TH vs. dG2/dG2;WT/TH and A30P vs. dG2/dG2;A30P/TH and A53T vs. dG2/dG2;A53T/TH). ^***^ represents *p* < 0.0001, ^**^p < 0.001, and ^*^p < 0.05.

Sleep disturbances and changes in sleep behavior appear much earlier preceding all other features in PD (Schapira, 2004; Arnulf et al., 2008). Common sleep problems associated with PD is excessive day-time sleepiness, Rapid eye movement (REM), night-time wakefulness and restless legs syndrome. Hence we analyzed sleep like rest behavior (defined as absence of movement for 5 consecutive minutes) in 0–3 day old young flies as well as in 30-day old flies using activity monitor. Decreased sleep (especially in the night), with increased number of sleep bouts (also known as sleep fragments) and decreased wake activity, in general, can stand as markers for PD pathogenicity in a fly model. It is noteworthy that sleep is only partially regulated by circadian rhythm, and other environmental factors and stress play critical role (Koh et al., 2006). Silencing of GBA1 gene in young flies expressing WT/A30P/A53T mutant synuclein under TH-Gal4 driver has profound effect on sleep-like rest behavior in flies. GBA1 silencing *per se* caused slight decrease daytime sleep in control flies (Figure 6A) as already reported in another study (Kawasaki et al., 2017) and we found an increase in number of sleep bouts in GBA1 silenced control as well as WT, A30P, & A53T α-syn expressing young flies (Figures 6A,B). GBA1 silencing *per se* also caused a decreased overall activity in flies (Figure 6C). Thus, GBA silenced α-syn expressing young did not show marked sleep pathology. However, in 30 day old flies, α-syn mutant A53T expression in GBA silenced flies show marked sleep pathology. Similar to young flies, 30 day old dG2/dG2 control flies show increased sleep fragments in day (Figure 6D) and decreased wake activity during day (Figure 6F) and GBA silencing *per se* causes decreased average sleep bout length during night that's observed in GBA silenced α-syn wildtype and mutant expressing flies also (Figure 6E). Similar to dG2/dG2 control flies, the GBA silenced A53T expressing 30 day old flies show increased number of sleep bouts in day (Figure 6D), decreased total sleep time and sleep bout length during night (Figure 6E) and decreased wake activity during day, when compared to their respective A53T control flies. Nevertheless, the expression of A53T mutant in these GBA silenced flies intensifies the sleep pathology. A53T expression in GBA silenced old flies causes decreased wake activity during day, night and in total when compared to both dG2/dG2 control and A53T control flies (Figure 6F). Although we observe a trend in sleep when GBA is knocked down in young flies, (the GBA knocked down young flies had an increased sleep bout and decreased overall activity) in old flies, only dG2/dG2 A53T fly shows a significant decline in activity while the opposite happens with dG2/dG2 WT flies. Such variation between young and old flies and between flies expressing WT and mutant α-syn can be due to the dynamic nature of synuclein that affects these features in flies (Gajula Balija et al., 2011).

## Discussion

In the current study, we aimed to explore the effect of reduced GCase enzyme activity on the aggregation and toxicity of human WT α-syn or its mutants (A30P and A53T α-syn). We obtained flies with GBA silencing in the presence or absence of WT, A30P and A53T α-syn and studied α-syn aggregation potential, neurodegeneration, climbing ability, locomotor activity and non-motor features like sleep behavior in these flies. These flies presumably have ~50% reduction in GCase activity thus depicting heterozygous GBA1 mutations associated with PD. We chose to compare WT α-Syn with A30P & A53T point mutants since A30P has been shown to aggregate much later than WT α-syn due to impaired β-structure while A53T has been shown to form both soluble oligomers and insoluble fibrils more rapidly (Karpinar et al., 2009). Although A53T GBA RNAi flies show a slight decrease in triton-insoluble fraction compared to A53T control flies at 0 days, there is a significant increase at 30 days indicating that aggregation increases in the absence of significant GCase activity as they age in A53T flies. This trend is not seen in WT α-Syn or A30P GBA RNAi flies. Thus western blot analysis revealed that the monomeric α-syn is lower with corresponding increase in α-syn aggregates in lysates of flies with GBA-RNAi co-expressed with A53T compared to control A53T flies. This finding was further confirmed by confocal staining using conformational antibody for α-syn (anti-α-synuclein filament antibody) where A53T GBA RNAi flies show more neuronal aggregates than that of A53T flies without GBA RNAi. Although western blot results using GMR driver is a strong evidence to conclude the finding, we did confocal imaging for syn aggregate using TH driver to support the finding. However, elaborate study is necessary to evaluate the presence of aggregated syn in different cluster and co-localization with non-aggregated synuclein antibody. With the absence of significant differences in the mRNA levels (Figure 2), we are tempted to speculate that α-syn with A53T mutation undergoes a physiological change during reduced GCase enzyme activity, possibly due to decreased autophagic flux (Davis et al., 2016) forming toxic oligomeric species or high molecular aggregates.

Since the presence of insoluble aggregates may not always contribute to increased toxicity (Kawasaki et al., 2017), we studied the neurodegeneration in these flies by TH neuron staining of fly brain. Confocal microscopy revealed a significant neurodegeneration in the order of A53>A30P>WT α-syn expressing aged flies. Neurodegeneration was aggravated in GBA1 RNAi flies expressing WT/A30P/A53T α-syn compared to control flies that expresses only WT/A30P/A53T α-syn, strongly suggesting that reduced GCase activity enhances DA neuronal loss and hence the toxicity of α-syn. Similar decrease in neuron cluster and neurodegeneration has been reported during GBA silencing in *drosophila* expressing WT α-syn (Davis et al., 2016). It has been proposed that α-syn clearance can take place in the lysosomes, and because of impaired lysosomal activity resulting from mutation in GBA, there is a buildup of misfolded forms of α-syn, further contributing to intracellular toxicity. Recent reports suggest that there is a mild accumulation of α-syn during knock-down of GBA in primary neuronal culture. Human induced-pluripotent-stem-cell-derived neurons generated from GD patients fibroblasts had an accumulation of α-syn that resulted in neurotoxicity that can be attributed to aggregation-dependent mechanisms (Mazzulli et al., 2011). It has been suggested that increased concentrations of lysosomal glucosylceramides resulting from diminished enzymatic activity promoted the formation of soluble α-synuclein oligomers and fibrils. Further it is indicated that increased concentrations of cytoplasmic α-syn blocked endoplasmic reticulum–Golgi apparatus trafficking of glucocerebrosidase, thus forming a bi-directional loop by decreasing the lysosomal glucocerebrosidase and thus causing increase in glucosylceramide and stable α-syn oligomers. In the current study we noticed that although there is no significant accumulation of α-syn during GBA knockdown, it aggravates aggregation of mutant α-syn A53T.

Patients with GBA mutation associated with PD tend to have an earlier age of onset and associated with cognitive decline and dementia. Motor impairments in PD patients is often preceded by non-motor features such as sleep problems. Sleep abnormalities in PD patients can be diagnosed years before motor syndromes appear (Schapira, 2004; Arnulf et al., 2008). GBA mutations are reported to be associated with Rapid eye movement (REM) sleep disorder (Gan-Or et al., 2015) which acts as early clinical sign for the development of PD and Lewybody associated dementia (DLB). It has been already reported in *drosophila* that the expression of pre-fibrillar α-syn mutants like TP-α-syn causes non-motor features like an altered sleep-like rest behavior that is observed prior to cell death, suggesting an early impairment of the DA system (Gajula Balija et al., 2011). Our study reveals that mutant A53T expression in GBA silenced old flies causes marked decline in their wake activity when studied using *drosophila* activity monitor (DAM). This pathology can be correlated to an increase in aggregation A53T mutant GBA RNAi flies that leads to increased DA neuronal loss. Our results on negative geotaxis assay indicate that GBA silencing *per se* causes decreased locomotor activity, similar to previous reports (Fishbein et al., 2014). We also observed a decreased locomotor activity when GBA is silenced in young flies. However, expression of mutant α-syn A53T during GBA silencing in old flies causes marked decrease in locomotor activity.

The current study shows that A53T mutants due to possible changes in metabolism during GBA silencing, forms more toxic aggregates (as evidenced by both western blot) leading to increased DA neuronal loss, affecting locomotor activity in these flies. More interestingly, these flies also exhibited an altered sleep behavior during GBA silencing. Our results show that the increase in α-syn aggregation and neurodegeneration in A53T flies with reduced GCase activity may be due to an altered α-syn metabolism. In conclusion, this study demonstrates that the reduced GCase activity both in the context of heterozygous GBA1 mutation associated with PD and in old age, may contribute to increased aggregation of mutant α-syn A53T and exacerbates the phenotype in a fly model of PD.

## Author contributions

All the authors provided important intellectual content, reviewed the content and approved the final version for the manuscript. Contributed significantly, read and approved the manuscript: SA, ND, MH, MA, WC, and YY. Conceived and designed and coordinated the experiments: MH. Performed the experiments: SB, ND, and MA. Analyzed the data: SB, ND, and MH. Contributed reagents, materials, analysis tools: MH, WC, and YY. Wrote the paper: SB, ND, and MH.

### Conflict of interest statement

The authors declare that the research was conducted in the absence of any commercial or financial relationships that could be construed as a potential conflict of interest.

## Supplementary material

Figure S1 Representative eye pictures showing no phenotypic effect in male and female flies maintained at 25°C carrying two copies of dG2 (GBA RNAi) with single copy of GMR-Gal4 driver expressing WT, A30P and A53T α-syn compared to fly eyes carrying single copy of GMR-Gal4 alone.

## Abbreviations

GBA: β-glucocerebrosidase
PD: Parkinson's disease
DAM: *Drosophila* activity monitor
PPL1: posterior protocerebral lateral 1
DA: Dopamine
SNc: Substantia nigra pars-compacta
α-syn: α-synuclein
GlcCer: Glucosylceramide
GD: Gaucher's disease
WT: Wildtype
dGBA1: *drosophila* homolog of GBA1
PBS-T: Phosphate buffered saline-Tween20
LD: Light Dark
TH: Tyrosine Hydroxylase
DLB: Lewybody associated dementia
iPSc: induced pluripotent stem cells.

## References

[B1] Aflaki E. Borger D. K. Moaven N. Stubblefield B. K. Rogers S. A. Patnaik S.. (2016). A new glucocerebrosidase chaperone reduces α-synuclein and glycolipid levels in iPSC-derived dopaminergic neurons from patients with gaucher disease and Parkinsonism. J. Neurosci. 36, 7441–7452. 10.1523/JNEUROSCI.0636-16.2016 27413154 PMC4945664

[B2] Arnulf I. Leu S. Oudiette D. (2008). Abnormal sleep and sleepiness in Parkinson's disease. Curr. Opin. Neurol. 21, 472–477. 10.1097/WCO.0b013e328305044d 18607209

[B3] Barkhuizen M. Anderson D. G. Grobler A. F. (2016). Advances in GBA-associated Parkinson's disease–Pathology, presentation and therapies. Neurochem. Int. 93, 6–25. 10.1016/j.neuint.2015.12.004 26743617

[B4] Bartels T. Choi J. G. Selkoe D. J. (2011). α-Synuclein occurs physiologically as a helically folded tetramer that resist aggregation. Nature 477, 107–110. 10.1038/nature10324 21841800 PMC3166366

[B5] Braak H. Braak E. (2000). Pathoanatomy of Parkinsons disease. J. Neurol. 247(Suppl. II), II3–II10. 10.1007/PL00007758 10991663

[B6] Brand A. H. Perrimon N. (1993). Targeted gene expression as a means of altering cell fates and generating dominant phenotypes. Development 118, 401–415. 8223268 10.1242/dev.118.2.401

[B7] Conway K. A. Harper J. D. Lansbury P. T. Jr. (2000a). Fibrils formed *in vitro* from alpha-synuclein and two mutant forms linked to Parkinson's disease are typical amyloid. Biochemistry 39, 2552–2563. 10.1021/bi991447r 10704204

[B8] Conway K. A. Lee S. J. Rochet J. C. Ding T. T. Williamson R. E. Lansbury P. T. (2000b). Acceleration of oligomerization, not fibrillization, is a shared property of both alpha-synuclein mutations linked to early-onset Parkinson's disease: implications for pathogenesis and therapy. Proc. Natl. Acad. Sci. U.S.A. 97, 571–576. 10.1073/pnas.97.2.571 10639120 PMC15371

[B9] Cullen V. Sardi S. P. Ng J. Xu Y. H. Sun Y. Tomlinson J. J.. (2011). Acid beta-glucosidase mutants linked to Gaucher disease, Parkinson disease, and Lewy body dementia alter alpha-synuclein processing. Ann. Neurol. 69, 940–953. 10.1002/ana.22400 21472771

[B10] Davis M. Y. Trinh K. Thomas R. E. Yu S. Germanos A. A. Whitley B. N.. (2016). Glucocerebrosidase deficiency in *Drosophila* Results in alpha-synuclein-independent protein aggregation and neurodegeneration. PLoS Genet. 12:e1005944. 10.1371/journal.pgen.1005944 27019408 PMC4809718

[B11] Feany M. B. Bender W. W. (2000). A *Drosophila* model of Parkinson's disease. Nature 404, 394–398. 10.1038/35006074 10746727

[B12] Fishbein I. Kuo Y. M. Giasson B. I. Nussbaum R. L. (2014). Augmentation of phenotype in a transgenic Parkinson mouse heterozygous for a Gaucher mutation. Brain 137, 3235–3247. 10.1093/brain/awu291 25351739 PMC4240298

[B13] Friggi-Grelin F. Coulom H. Meller M. Gomez D. Hirsh J. Birman S. (2003). Targeted gene expression in *Drosophila* dopaminergic cells using regulatory sequences from tyrosine hydroxylase. J. Neurobiol. 54, 618–627. 10.1002/neu.10185 12555273

[B14] Gajula Balija M. B. Griesinger C. Herzig A. Zweckstetter M. Jäckle H. (2011). Pre-fibrillar alpha-synuclein mutants cause Parkinson's disease-like non-motor symptoms in *Drosophila*. PLoS ONE 6:e24701. 10.1371/journal.pone.0024701 21931820 PMC3169624

[B15] Gan-Or Z. Mirelman A. Postuma R. B. Arnulf I. Bar-Shira A. Dauvilliers Y.. (2015). GBA mutations are associated with rapid eye movement sleep behavior disorder. Ann. Clin. Transl. Neurol. 2, 941–945. 10.1002/acn3.228 26401515 PMC4574811

[B16] Gegg M. E. Burke D. Heales S. J. Cooper J. M. Hardy J. Wood N. W.. (2012). Glucocerebrosidase deficiency in substantia nigra of parkinson disease brains. Ann. Neurol 72, 455–463. 10.1002/ana.23614 23034917 PMC3638323

[B17] Gibb W. R. Lees A. J. (1991). Anatomy, pigmentation, ventral and dorsal subpopulations of the substantia nigra, and differential cell death in Parkinson's disease. J. Neurol. Neurosurg. Psychiatry 54, 388–396. 10.1136/jnnp.54.5.388 1865199 PMC488535

[B18] Goker-Alpan O. Schiffmann R. LaMarca M. E. Nussbaum R. L. McInerney-Leo A. Sidransky E. (2004). Parkinsonism among Gaucher disease carriers. J. Med. Genet. 41, 937–940. 10.1136/jmg.2004.024455 15591280 PMC1735652

[B19] Greenbaum E. A. Graves C. L. Mishizen-Eberz A. J. Lupoli M. A. Lynch D. R. Englander S. W.. (2005). The E46K mutation in alpha-synuclein increases amyloid fibril formation. J. Biol. Chem. 280, 7800–7807. 10.1074/jbc.M411638200 15632170

[B20] Joiner W. J. Crocker A. White B. H. Sehgal A. (2006). Sleep in *Drosophila* is regulated by adult mushroom bodies. Nature 441, 757–760. 10.1038/nature04811 16760980

[B21] Karpinar D. P. Balija M. B. Kügler S. Opazo F. Rezaei-Ghaleh N. Wender N.. (2009). Pre-fibrillar alpha-synuclein variants with impaired beta-structure increase neurotoxicity in Parkinson's disease models. EMBO J. 28, 3256–3268. 10.1038/emboj.2009.257 19745811 PMC2771093

[B22] Kawasaki H. Suzuki T. Ito K. Takahara T. Goto-Inoue N. Setou M.. (2017). Minos-insertion mutant of the *Drosophila* GBA gene homologue showed abnormal phenotypes of climbing ability, sleep and life span with accumulation of hydroxy-glucocerebroside. Gene 614, 49–55. 10.1016/j.gene.2017.03.004 28286087

[B23] Koh K. Evans J. M. Hendricks J. C. Sehgal A. (2006). A *drosophila* model for age-associated changes in sleep:wake cycles. Proc. Natl. Acad. Sci. U.S.A. 103, 13843–13847. 10.1073/pnas.0605903103 16938867 PMC1564207

[B24] Kraoua I. Sedel F. Caillaud C. Froissart R. Stirnemann J. Chaurand G.. (2011). A French experience of type 3 Gaucher disease: Phenotypic diversity and neurological outcome of 10 patients. Brain Dev. 33, 131–139. 10.1016/j.braindev.2010.02.005 20307947

[B25] Krüger R. Kuhn W. Müller T. Woitalla D. Graeber M. Kösel S.. (1998). Ala30Pro mutation in the gene encoding alpha-synuclein in Parkinson's disease. Nat. Genet. 18, 106–108. 10.1038/ng0298-106 9462735

[B26] Mazzulli J. R. Xu Y. H. Sun Y. Knight A. L. McLean P. J. Caldwell G. A.. (2011). Gaucher disease glucocerebrosidase and alpha-synuclein form a bidirectional pathogenic loop in synucleinopathies. Cell 146, 37–52. 10.1016/j.cell.2011.06.001 21700325 PMC3132082

[B27] Murphy K. E. Halliday G. M. (2014). Glucocerebrosidase deficits in sporadic Parkinson disease. Autophagy 10, 1350–1351. 10.4161/auto.29074 24915553 PMC4203563

[B28] Murphy K. E. Gysbers A. M. Abbott S. K. Tayebi N. Kim W. S. Sidransky E.. (2014). Reduced glucocerebrosidase is associated with increased alpha-synuclein in sporadic Parkinson's disease. Brain 137, 834–848. 10.1093/brain/awt367 24477431 PMC3927701

[B29] Nuzhnyi E. Emelyanov A. Boukina T. Usenko T. Yakimovskii A. Zakharova E.. (2015). Plasma oligomeric alpha-synuclein is associated with glucocerebrosidase activity in Gaucher disease. Mov. Disord 30, 989–991. 10.1002/mds.26200 25962734

[B30] Pitman J. L. McGill J. J. Keegan K. P. Allada R. (2006). A dynamic role for the mushroom bodies in promoting sleep in *Drosophila*. Nature 441, 753–756. 10.1038/nature04739 16760979

[B31] Polymeropoulos M. H. Lavedan C. Leroy E. Ide S. E. Dehejia A. Dutra A.. (1997). Mutation in the alpha-synuclein gene identified in families with Parkinson's disease. Science 276, 2045–2047. 10.1126/science.276.5321.2045 9197268

[B32] Rocha E. M. Smith G. A. Park E. Cao H. Brown E. Hayes M. A.. (2015). Glucocerebrosidase gene therapy prevents alpha-synucleinopathy of midbrain dopamine neurons. Neurobiol. Dis. 82, 495–503. 10.1016/j.nbd.2015.09.009 26392287

[B33] Sardi S. P. Clarke J. Kinnecom C. Tamsett T. J. Li L. Stanek L. M.. (2011). CNS expression of glucocerebrosidase corrects alpha-synuclein pathology and memory in a mouse model of Gaucher-related synucleinopathy. Proc. Natl. Acad. Sci. U.S.A. 108, 12101–12106. 10.1073/pnas.1108197108 21730160 PMC3141921

[B34] Schapira A. H. (2004). Excessive daytime sleepiness in Parkinson's disease. Neurology 63(Suppl. 3), S24–S27. 10.1212/WNL.63.8_suppl_3.S24 15505139

[B35] Schapira A. H. (2015). Glucocerebrosidase and Parkinson disease: recent advances. Mol. Cell Neurosci. 66, 37–42. 10.1016/j.mcn.2015.03.013 25802027 PMC4471139

[B36] Shaw P. J. Cirelli C. Greenspan R. J. Tononi G. (2000). Correlates of sleep and waking in *Drosophila* melanogaster. Science 287, 1834–1837. 10.1126/science.287.5459.1834 10710313

[B37] Sidransky E. (2005). Gaucher disease and parkinsonism. Mol. Genet. Metab. 84, 302–304. 10.1016/j.ymgme.2004.11.007 15781189

[B38] Sidransky E. Hart P. S. (2012). Penetrance of PD in Glucocerebrosidase gene mutation carriers. Neurology 79, 106–107. 10.1212/01.wnl.0000416261.29035.4c 22753448

[B39] Sidransky E. Lopez G. (2012). The link between the GBA gene and parkinsonism. Lancet Neurol. 11, 986–998. 10.1016/S1474-4422(12)70190-4 23079555 PMC4141416

[B40] Suzuki M. Fujikake N. Takeuchi T. Kohyama-Koganeya A. Nakajima K. Hirabayashi Y.. (2015). Glucocerebrosidase deficiency accelerates the accumulation of proteinase K-resistant alpha-synuclein and aggravates neurodegeneration in a *Drosophila* model of Parkinson's disease. Hum. Mol. Genet 24, 6675–6686. 10.1093/hmg/ddv372 26362253

[B41] Whitworth A. J. Theodore D. A. Greene J. C. Benes H. Wes P. D. Pallanck L. J. (2005). Increased glutathione S-transferase activity rescues dopaminergic neuron loss in a *Drosophila* model of Parkinson's disease. Proc. Natl. Acad. Sci. U.S.A. 102, 8024–8029. 10.1073/pnas.0501078102 15911761 PMC1142368

