# Supplementary figures and images for: Silencing of Glucocerebrosidase Gene in Drosophila Enhances the Aggregation of Parkinson's Disease Associated α-Synuclein Mutant A53T and Affects Locomotor Activity
Source: Front Neurosci. 2018 Feb 16;12:81. doi: 10.3389/fnins.2018.00081 (PMC5820349; doi:10.3389/fnins.2018.00081)

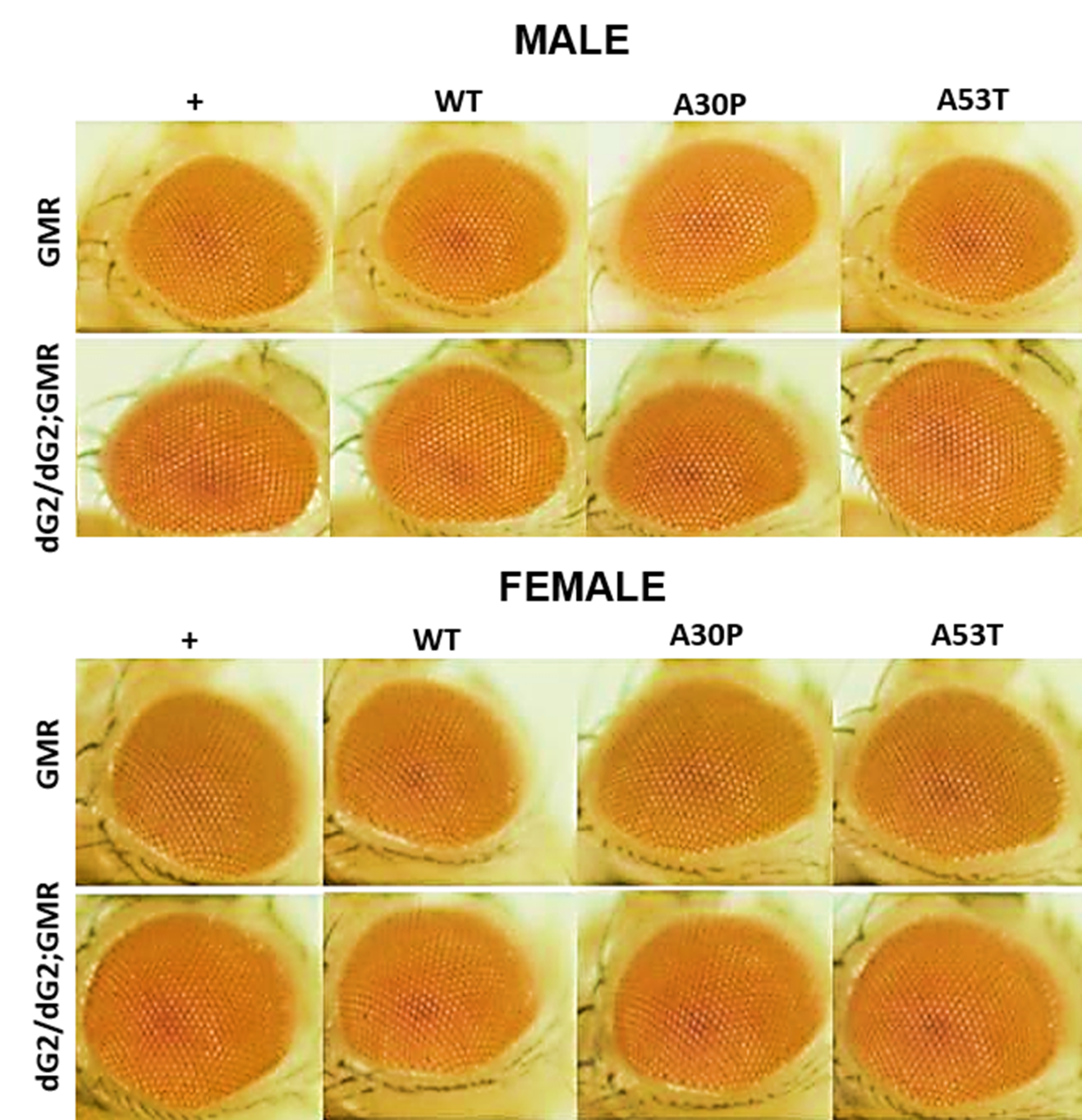

Supplement: Figure S1 — Representative eye pictures showing no phenotypic effect in male and female flies maintained at 25°C carrying two copies of dG2 (GBA RNAi) with single copy of GMR-Gal4 driver expressing WT, A30P and A53T α-syn compared to fly eyes carrying single copy of GMR-Gal4 alone. [file Image1.TIF]
